# Avelumab in patients with previously treated metastatic Merkel cell carcinoma: long-term data and biomarker analyses from the single-arm phase 2 JAVELIN Merkel 200 trial

**DOI:** 10.1136/jitc-2020-000674

**Published:** 2020-05-15

**Authors:** Sandra P D'Angelo, Shailender Bhatia, Andrew S Brohl, Omid Hamid, Janice M Mehnert, Patrick Terheyden, Kent C Shih, Isaac Brownell, Celeste Lebbé, Karl D Lewis, Gerald P Linette, Michele Milella, Sara Georges, Parantu Shah, Barbara Ellers-Lenz, Marcis Bajars, Gülseren Güzel, Paul T Nghiem

**Affiliations:** 1 Department of Medicine, Memorial Sloan Kettering Cancer Center, New York, New York, USA; 2 Department of Medicine, Weill Cornell Medical College, New York, New York, USA; 3 Department of Medicine, University of Washington Medical Center, Seattle, Washington, USA; 4 Sarcoma Department and Cutaneous Oncology, Moffitt Cancer Center, Tampa, Florida, USA; 5 Department of Medical Oncology, The Angeles Clinic and Research Institute, Los Angeles, California, USA; 6 Division of Medical Oncology, Rutgers Cancer Institute of New Jersey, New Brunswick, New Jersey, USA; 7 Department of Dermatology, University of Lübeck, Lübeck, Germany; 8 Department of Medical Oncology, Sarah Cannon Research Institute, Nashville, Tennessee, USA; 9 Department of Medical Oncology, Tennessee Oncology, Nashville, Tennessee, USA; 10 Dermatology Branch, NIH, Bethesda, Maryland, USA; 11 Dermatologie, Université de Paris, INSERM U976, Paris, France; 12 Dermatology and CIC, AP-HP, Saint Louis Hospital, Paris, France; 13 Department of Medicine, University of Colorado Denver School of Medicine, Aurora, Colorado, USA; 14 Center for Cellular Immunotherapies, University of Pennsylvania, Philadelphia, Pennsylvania, USA; 15 Department of Medical Oncology, IRCCS Regina Elena National Cancer Institute, Rome, Italy; 16 Clinical Biomarkers and Companion Diagnostics, Department of Translational Medicine, EMD Serono Research & Development Institute, Billerica, Massachusetts, USA; 17 Bioinformatics, Department of Translational Medicine, EMD Serono Research & Development Institute, Billerica, Massachusetts, USA; 18 Global Biostatistics, Merck KGaA, Darmstadt, Germany; 19 Clinical Development, EMD Serono Research & Development Institute, Billerica, Massachusetts, USA; 20 Clinical Development, Merck KGaA, Darmstadt, Germany; 21 Division of Dermatology, Department of Medicine, University of Washington Medical Center at South Lake Union, Seattle, Washington, USA

**Keywords:** skin neoplasms, clinical trials, phase II as topic, biomarkers, tumor, immunotherapy

## Abstract

**Background:**

Merkel cell carcinoma (MCC) is a rare, aggressive skin cancer associated with a high risk of metastasis. In 2017, avelumab (anti–programmed death-ligand 1 (PD-L1)) became the first approved treatment for patients with metastatic MCC (mMCC), based on the occurrence of durable responses in a subset of patients. Here, we report long-term efficacy and safety data and exploratory biomarker analyses in patients with mMCC treated with avelumab.

**Methods:**

In a cohort of this single-arm, phase 2 trial (JAVELIN Merkel 200), patients with mMCC and disease progression after prior chemotherapy received avelumab 10 mg/kg intravenously every 2 weeks. The primary endpoint was confirmed objective response rate (ORR) by independent review per Response Evaluation Criteria in Solid Tumors V.1.1. Other assessments included duration of response, progression-free survival, overall survival (OS), safety and biomarker analyses.

**Results:**

As of 14 September 2018, 88 patients had been followed up for a median of 40.8 months (range 36.4–49.7 months). The ORR was 33.0% (95% CI 23.3% to 43.8%), including a complete response in 11.4% (10 patients), and the median duration of response was 40.5 months (95% CI 18.0 months to not estimable). As of 2 May 2019 (≥44 months of follow-up), the median OS was 12.6 months (95% CI 7.5 to 17.1 months) and the 42-month OS rate was 31% (95% CI 22% to 41%). Of long-term survivors (OS >36 months) evaluable for PD-L1 expression status (n=22), 81.8% had PD-L1+ tumors. In exploratory biomarker analyses, high tumor mutational burden (≥2 non-synonymous somatic variants per megabase) and high major histocompatibility complex class I expression (30% of tumors with highest expression) were associated with trends for improved ORR and OS. In long-term safety assessments (≥36 months of follow-up), no new or unexpected adverse events were reported, and no treatment-related deaths occurred.

**Conclusions:**

Avelumab showed continued durable responses and meaningful long-term survival outcomes in patients with mMCC, reinforcing avelumab as a standard-of-care treatment option for this disease.

**Trial registration number:**

NCT02155647

## Background

Merkel cell carcinoma (MCC) is a rare, aggressive skin cancer associated with excessive sun exposure, immunosuppression and the presence of clonally integrated Merkel cell polyomavirus (MCPyV).^1^ Patients with metastatic MCC (mMCC) have a poor prognosis, with a historical 5-year overall survival (OS) rate of ≤18%.^1–3^ MCC is considered chemosensitive, and cytotoxic chemotherapy achieves relatively high objective response rates (ORRs); however, patients typically have transient responses, limited survival and experience considerable toxicity.^2 4–6^

Antibodies that target the programmed death-ligand 1 (PD-L1)/programmed cell death-1 (PD-1) immune checkpoint have shown unprecedented clinical activity in mMCC and induce durable responses in a subset of patients.^7–11^ Avelumab is a human anti–PD-L1 IgG1 monoclonal antibody that has received regulatory approval in multiple countries for the treatment of mMCC based on results from the phase 2 JAVELIN Merkel 200 clinical trial. Preclinical studies have suggested that in addition to stimulating adaptive immune responses against tumor cells, avelumab may also engage innate effector cell functions through its wild-type Fc region, unlike other approved anti–PD-L1/PD-1 antibodies.^12–14^

Previously, results from patients with mMCC enrolled in JAVELIN Merkel 200 who had disease progression after ≥1 prior line of chemotherapy were reported from the primary analysis and after ≥1 year of follow-up.^9 10^ We report efficacy and safety with ≥36 months of follow-up, OS analyses with ≥44 months of follow-up, and exploratory biomarker analyses with ≥24 months of follow-up.

## Methods

### Study design and participants

The design of JAVELIN Merkel 200, a phase 2, prospective, single-arm, open-label, multicenter trial (NCT02155647), was reported previously.^9 10^ Briefly, eligible patients were aged ≥18 years and had an Eastern Cooperative Oncology Group performance status (ECOG PS) of 0–1; histologically confirmed, measurable (per Response Evaluation Criteria in Solid Tumors (RECIST) V.1.1) stage IV MCC that had progressed following ≥1 prior line of chemotherapy for metastatic disease; and adequate hematologic, hepatic and renal function. Patients were ineligible if they had received previous immune checkpoint inhibitor therapy, were receiving concurrent anticancer treatment or systemic treatment with corticosteroids or had immunosuppression or other clinically significant comorbidities.

### Procedures

Patients received avelumab 10 mg/kg by 1-hour intravenous infusion every 2 weeks until confirmed disease progression, unacceptable toxicity or other criteria for withdrawal occurred.^9^ Patients received premedication with antihistamine (eg, diphenhydramine) and acetaminophen, per local treatment standards, 30–60 min before each infusion. Tumors were assessed radiologically every 6 weeks according to RECIST 1.1, adjudicated by an independent review committee (IRC). Patients who had a confirmed complete response (CR) received subsequent treatment for ≥6 months and could then withdraw from treatment per investigator discretion and in observance of withdrawal criteria. Treatment beyond 12 months post confirmed CR was allowed per investigator judgment. Patients could remain on treatment beyond disease progression based on clinical judgment, provided there was no significant clinical deterioration, defined as no new/worsening symptoms, no change in ECOG PS to ≥3 for >14 days and no requirement for salvage therapy.

Adverse events (AEs) were assessed according to National Cancer Institute Common Terminology Criteria for Adverse Events (NCI-CTCAE) V.4.0. Immune-related AEs (irAEs) were identified using a prespecified list of Medical Dictionary for Regulatory Activities (MedDRA) Preferred Terms, followed by comprehensive medical review. Infusion-related reactions (IRRs) were assessed using an expanded definition that included events occurring on the day of or day after infusion and signs/symptoms occurring on the day of infusion (during or after the infusion) that resolved on the day of onset or the next day, based on a prespecified list of MedDRA Preferred Terms.

Biomarker analyses used formalin-fixed, paraffin-embedded tumor samples obtained from the metastatic site (preferred) or primary tumor. PD-L1 expression by tumor cells was measured using the PD-L1 73-10 immunohistochemistry (IHC) assay (Dako, Carpenteria, California, USA). PD-L1 positivity was defined as PD-L1 expression in ≥1% of tumor cells. MCPyV status was determined by real-time PCR using DNA extracted from tumor samples, TaqMan (Thermo Fisher Scientific, Waltham, Massachusetts, USA) reagents, and small T-antigen–specific primers, and by IHC using a mouse monoclonal antibody (clone CM2B4; Santa Cruz Biotechnology, Dallas, Texas, USA). CD8 IHC was performed using a mouse monoclonal antibody (clone C8/144B; Dako) and evaluated by digital image analysis using Aperio Nuclear V.9 Algorithm (Leica Biosystems, Buffalo Grove, Illinois, USA). CD8+ T cell density was evaluated at the tumor invasive margin (from 500 µm outside to 500 µm inside the leading edge of the tumor in samples with an apparent tumor/normal boundary) and at the center of the tumor (beginning inside the inner invasive margin border and comprising the rest of the tumor, including intervening stromal bands).

To assess tumor mutational burden (TMB), the average number of non-synonymous somatic variants per megabase (NSSV/Mb) was calculated from patient-matched tumor and blood whole-exome sequencing profiles. Empirical TMB cut-offs of <2 NSSV/Mb (low) and ≥2 NSSV/Mb (high) were chosen based on the distribution of TMB values in this population and to include a sufficient number of patients per subgroup. Gene expression ranks of major histocompatibility complex (MHC) class I genes (HLA-A, HLA-B and HLA-C) were calculated using RNA sequencing data from normal tissue samples (Genotype-Tissue Expression (GTEx)) and patient tumor samples. Genome-wide copy number changes and loss of heterozygosity (LOH) at the HLA locus were analyzed using Sequenza^15^ and a modified version of OptiType.^16^ Gene set enrichment analysis (GSEA) was carried out on unselected gene signature lists using Hallmark and Reactome pathway gene sets from the Molecular Signatures Database^17 18^ and EMD Serono’s internal collection. A fast preranked GSEA package was used with ranked lists of genes between conditions. Results were filtered using a cut-off of a normalized enrichment score of 2 and a false discovery rate of <1%.

### Outcomes

Outcomes from the primary analysis and 1-year follow-up analysis have been reported previously.^9 10^ The primary endpoint was confirmed best overall response per RECIST 1.1 by IRC. Secondary endpoints included duration of response (DOR; time from CR or partial response (PR) until documented disease progression or death), response status, progression-free survival (PFS) per RECIST 1.1 by IRC, OS and safety. Exploratory analyses included shrinkage in target lesions and biomarker analyses.

### Assessments and statistical analysis

Clinical activity and safety were analyzed descriptively in all patients who received ≥1 dose of avelumab. ORRs were calculated with 2-sided 95% CIs using the Clopper-Pearson method. Time-to-event endpoints (PFS, OS and DOR) were estimated with the Kaplan-Meier method, and 95% CIs for the median were calculated using the Brookmeyer-Crowley method.

## Results

### Patients

Between 25 July 2014 and 3 September 2015, 88 patients were enrolled and treated with avelumab. All patients had received ≥1 prior line of systemic anticancer treatment (online supplementary file 1). After ≥36 months of follow-up (data cut-off, 14 September 2018), median follow-up was 40.8 months (range 36.4–49.7 months). Patients were treated for a median of 3.9 months (range 0.5–47.9 months); six patients (6.8%) received >3 years of treatment. After ≥44 months of follow-up (assessment of OS and subsequent therapy only; data cut-off, 2 May 2019), three patients (3.4%) were still receiving treatment. Treatment was discontinued in 85 patients (96.6%), most commonly for disease progression (44 (50.0%); online supplementary file 1).

10.1136/jitc-2020-000674.supp1 Supplementary data

### Antitumor activity

After ≥36 months of follow-up, confirmed objective responses to avelumab had occurred in 29 of 88 patients (33.0% (95% CI 23.3% to 43.8%)), including CR in 10 patients (11.4%) (table 1), which was unchanged from the 1-year analysis.^10^ In patients who had received 1 (n=52) vs ≥2 (n=36) prior systemic anticancer treatments in any disease stage, the ORRs were 40.4% (95% CI 27.0% to 54.9%) and 22.2% (95% CI 10.1% to 39.2%), respectively. In patients with (n=47) or without (n=41) visceral metastases at baseline, the ORRs were 34.0% (95% CI 20.9% to 49.3%) and 31.7% (95% CI 18.1% to 48.1%), respectively. In evaluable patients with PD-L1+ (n=57) or PD-L1− (n=16) tumors, the ORRs were 36.8% (95% CI 24.4% to 50.7%) and 18.8% (95% CI 4.0% to 45.6%), respectively. In evaluable patients with MCPyV+ (n=46) or MCPyV− (n=31) tumors, the ORRs were 28.3% (95% CI 16.0% to 43.5%) and 35.5% (95% CI 19.2% to 54.6%), respectively. Best change from baseline in target lesions is shown in online supplementary file 1.

**Table 1:** Objective responses to avelumab after ≥36 months of follow-up

Response	N=88
Confirmed best overall response, n (%)	
Complete response	10 (11.4)
Partial response	19 (21.6)
Stable disease	9 (10.2)
Progressive disease	32 (36.4)
Not evaluable	18 (20.5)
Objective response rate (95% CI), %	33.0 (23.3 to 43.8)
Disease control rate, %	43.2

Response durability	n=29
Median duration of response (95% CI), months	40.5 (18.0 to not estimable)
Range	2.8–41.5
Proportion with duration of response (95% CI), %	
≥6 months	93 (75 to 98)
≥1 year	71 (51 to 85)
≥2 years	67 (47 to 82)
≥3 years	52 (26 to 73)

**Figure 1:**
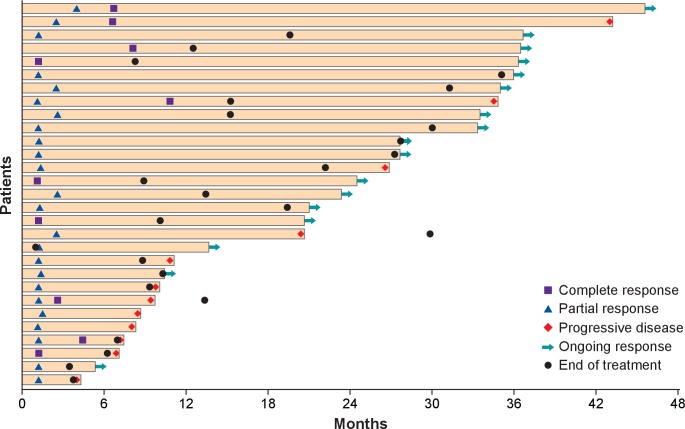
Time to and duration of response after ≥36 months of follow-up (n=29).

Responses were ongoing at last available tumor assessment in 17 of 29 responders (58.6%), including 5 of 10 with a CR. Four patients with continuing tumor assessments had an ongoing response lasting ≥3 years (figure 1), including one patient with an ongoing CR who had received 88 doses of avelumab. Median DOR was 40.5 months (95% CI 18.0 months to not estimable). The longest recorded DORs were 41.5 and 40.5 months in two patients with an ongoing CR. By Kaplan-Meier estimate, the proportion of responses with a duration ≥3 years was 52% (95% CI 26% to 73%). Long-term responses (≥3 years) were observed in patients with PD-L1+ and PD-L1− tumors (online supplementary file 1). Of 12 responders who subsequently had disease progression (including seven who discontinued avelumab before disease progression), progression was generally due to a new lesion rather than an increase in existing lesions. Time from start of treatment until progression was <12 months in eight patients. In the other four patients who progressed after >12 months, progression followed PR (patients were progression-free for 20.6 and 26.8 months) and CR (34.8 and 43.2 months). Of these four patients, two had discontinued avelumab before disease progression. Of 17 patients with an ongoing response at last follow-up, 16 had discontinued treatment (4 with CR and 12 with PR). In four patients who discontinued avelumab with an ongoing CR (permitted per protocol), DOR after discontinuation ranged from 10.2 to 27.6 months (overall DOR, 19.3–35.0 months).

Based on Kaplan-Meier analysis, PFS rates at 24 months and 36 months were 26% (95% CI 17% to 36%) and 21% (95% CI 12% to 32%), respectively.

### Overall survival

**Figure 2:**
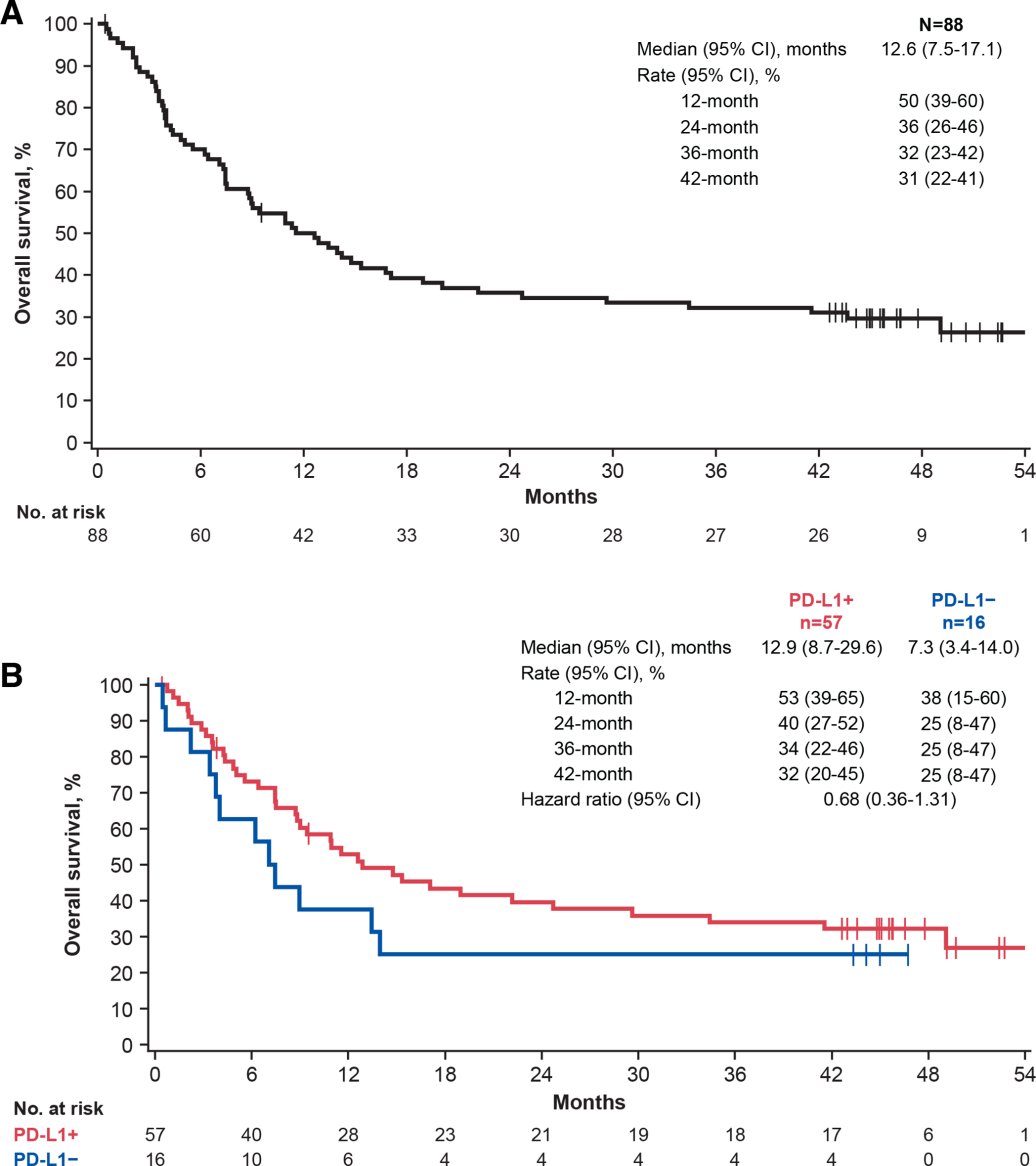
Overall survival with avelumab after ≥44 months of follow-up. (A) All patients. (B) Subgroups defined by PD-L1 status. PD-L1, programmed death-ligand 1.

OS was analyzed after ≥44 months of follow-up. Median OS was 12.6 months (95% CI 7.5 to 17.1 months), and OS rates at 36 and 42 months were 32% (95% CI 23% to 42%) and 31% (95% CI 22% to 41%), respectively (figure 2A). The longest recorded OS duration was 54.8 months (patient with CR). Median OS in patients with PD-L1+ (n=57) or PD-L1− (n=16) tumors was 12.9 months (95% CI 8.7 to 29.6 months) and 7.3 months (95% CI 3.4 to 14.0 months), respectively (figure 2B; HR, 0.68 (95% CI 0.36 to 1.31)).

### Subsequent therapy

Patients who had disease progression and discontinued avelumab continued to be followed up where possible (≥44-month data; online supplementary file 1). No durable responses were recorded with any subsequent therapy. Of 59 patients who did not have an objective response with avelumab (ie, refractory disease), 21 received a subsequent treatment, including radiotherapy (n=8), chemotherapy (n=10), another immune checkpoint inhibitor (n=5) or another systemic agent (n=6). A subsequent objective response occurred in four of these patients, all of whom had received chemotherapy. However, 20 of 21 patients who received any subsequent treatment had died by data cut-off, with one patient lost to follow-up. Among patients with refractory disease who had available data, death occurred within 1 year of disease progression on avelumab except for three patients who died within 2 years and one patient who died approximately 3 years later.

For patients who had an objective response with avelumab and subsequent disease progression (ie, acquired resistance), of 10 patients who initially had a CR, three received a subsequent therapy, namely radiotherapy (patient was alive approximately 1 year later), chemotherapy followed by nivolumab and ipilimumab then nivolumab alone (patient was alive approximately 3 years later) and chemotherapy (patient died within 2 months). Two patients with a PR withdrew from study treatment to receive commercial avelumab at another institution (for patient convenience) and were alive >2 years later.

### Exploratory biomarker analyses

**Figure 3:**
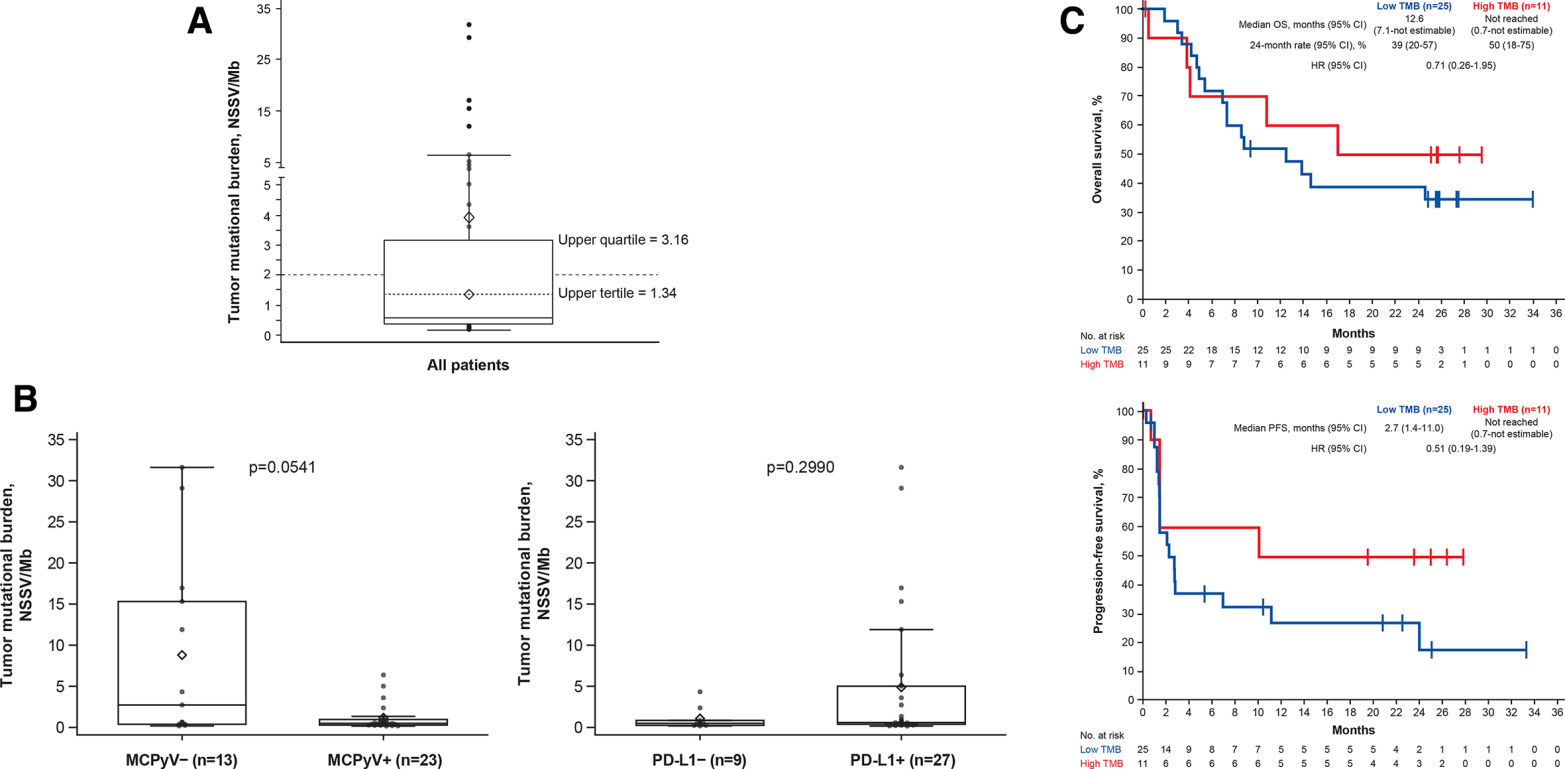
TMB in evaluable patients (n=36). (A) Distribution of values. (B) Association with viral and PD-L1 status. (C) OS and PFS by subgroup. The boxes represent IQRs, and the solid horizontal lines inside the boxes are medians. The upper whiskers denote the maximum observation below the upper fence, and the lower whiskers denote the minimum observation above the lower fence. The points outside the boxes are observations. Diamonds within boxes are the tertiles, and diamonds above boxes are the mean. p values were calculated using an exact Wilcoxon two-sample test. MCPyV, Merkel cell polyomavirus; NSSV/Mb, non-synonymous somatic variant per megabase; OS, overall survival; PD-L1, programmed death-ligand 1; PFS, progression-free survival; TMB, tumor mutational burden.

**Figure 4:**
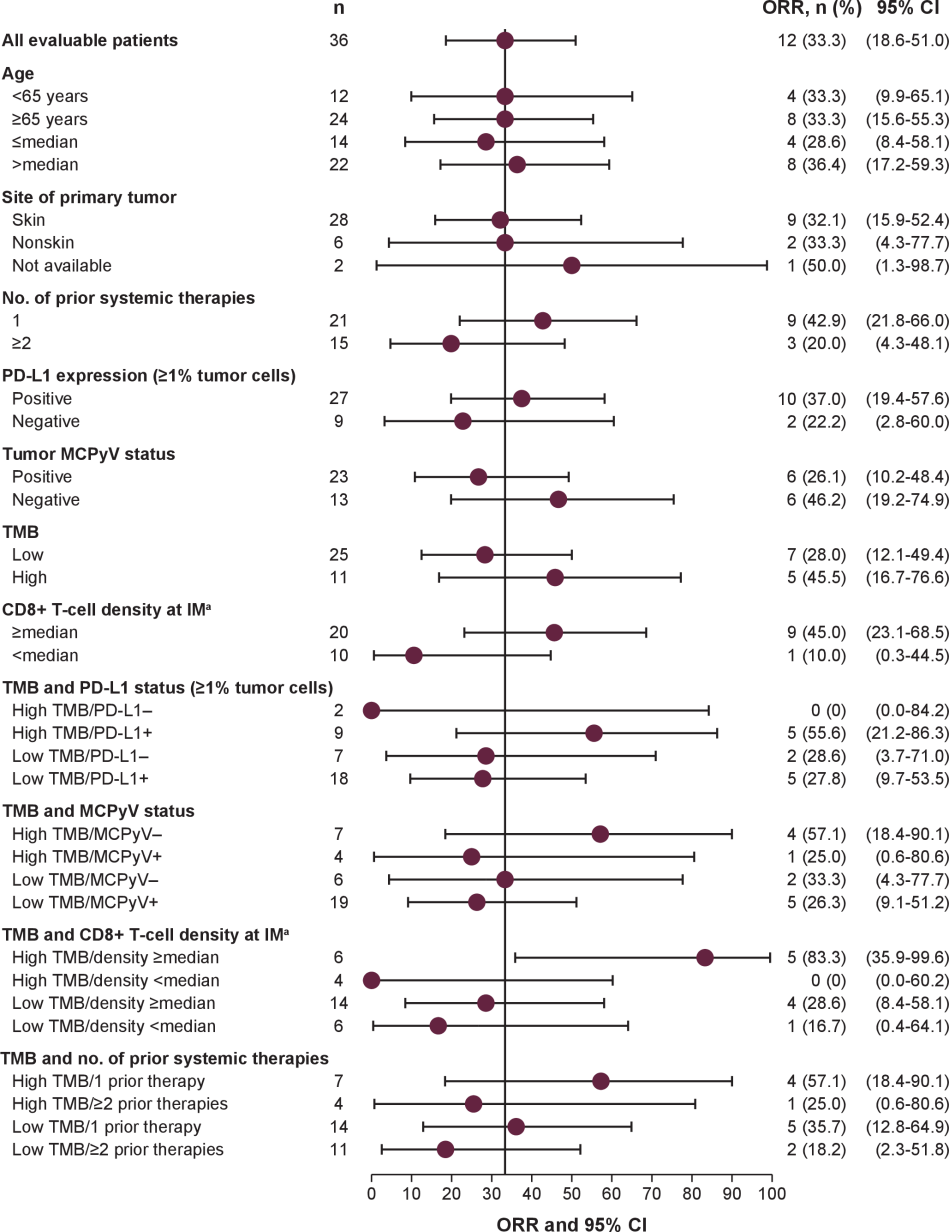
ORR in selected subgroups evaluable for TMB analysis. ^a^CD8+ T cell density data were missing for six patients. IM, invasive margin; MCPyV, Merkel cell polyomavirus; ORR, objective response rate; PD-L1, programmed death-ligand 1; TMB, tumor mutational burden.

Outcomes according to expression of exploratory biomarkers were analyzed after ≥24 months of follow-up (data cut-off, 24 September 2017). In 36 evaluable patients, median TMB was 0.58 NSSV/Mb (range 0.16–31.62 NSSV/Mb; figure 3A). Patients with MCPyV− vs MCPyV+ tumors had a trend for a higher median TMB (2.72 vs 0.49 NSSV/Mb; p=0.0541); in patients with PD-L1+ vs PD-L1− tumors, median TMB was 0.59 vs 0.49 NSSV/Mb (p=0.2990; figure 3B). Patients with a high vs low TMB (≥2 vs <2 NSSV/Mb) had ORRs of 45.5% (95% CI 16.7% to 76.6%) vs 28.0% (95% CI 12.1% to 49.4%; p=0.4455), 6-month PFS rates of 60.0% (95% CI 25% to 83%) vs 38.0% (95% CI 19% to 56%), and median OS not reached (95% CI 0.7 months to not estimable) vs 12.6 months (95% CI 7.1 to not estimable), respectively (figures 3C and 4). Among the exploratory subgroups, ORRs were highest in patients with tumors with high TMB that were also MCPyV− (57.1% (95% CI 18.4% to 90.1%)), PD-L1+ (55.6% (95% CI 21.2% to 86.3%)) or had a greater than median CD8+ T cell density at the invasive margin (83.3% (95% CI 35.9% to 99.6%)), and in patients with only one prior systemic anticancer treatment (57.1% (95% CI 18.4% to 90.1%)).

**Figure 5:**
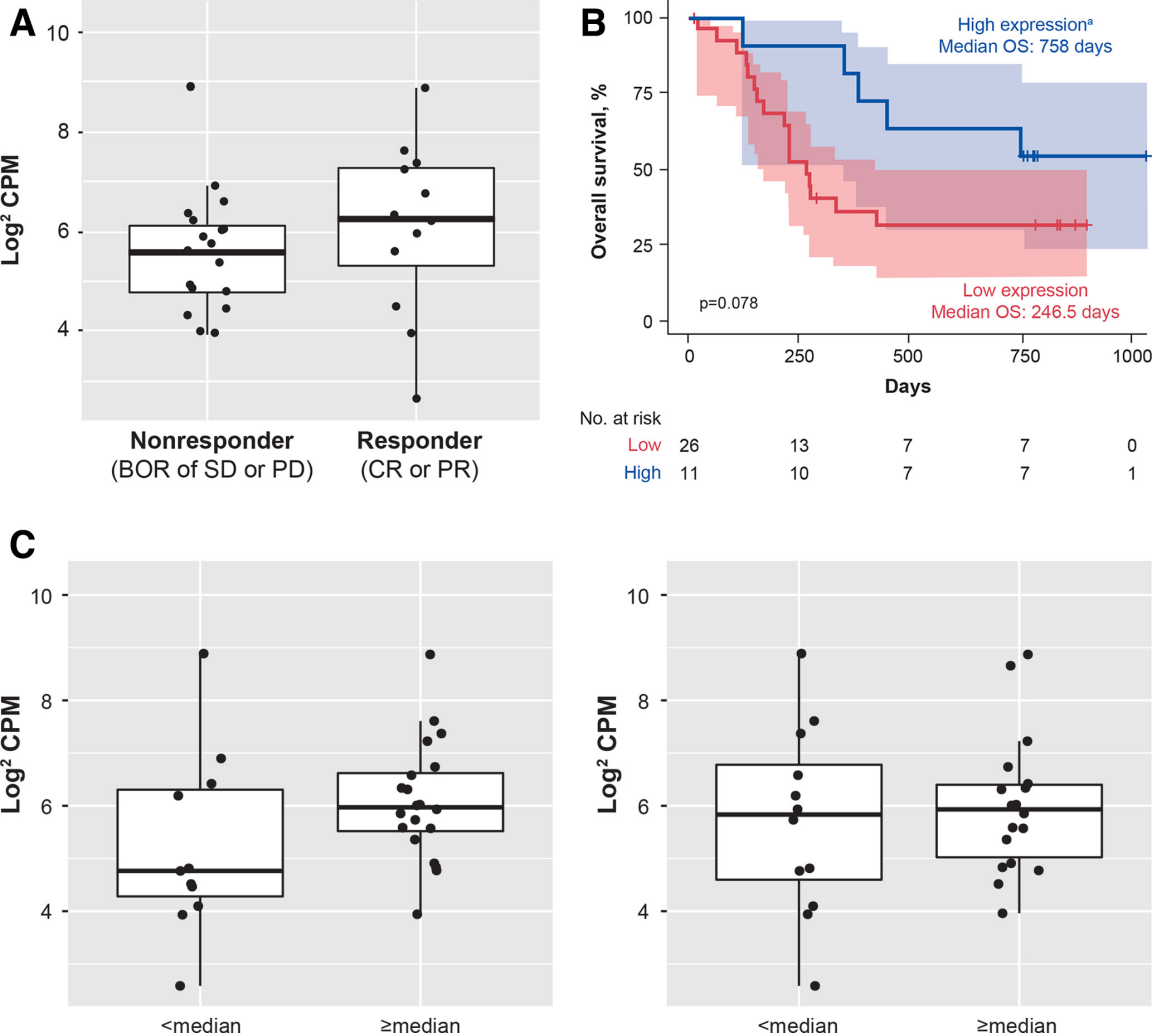
Association of MHC class I expression with (A) response, (B) OS (n=37) and (C) CD8+ T cell density expression in evaluable patients (n=31) at the IM (left) and tumor core (right). The boxes represent IQRs, and the horizontal lines are medians. The whiskers denote the lower and upper quartiles, and the circles represent data points. ^a^The high-expression subgroup was defined as patients in the top 30% of overall MHC expression. BOR, best overall response; CPM, count per million; CR, complete response; IM, invasive margin; MHC, major histocompatibility complex; OS, overall survival; PD, progressive disease; PR, partial response; SD, stable disease.

Expression of MHC class I HLA genes appeared to be downregulated in MCC tumors compared with normal tissues (online supplementary file 1). MHC class I genes were among the top 0.2% of genes expressed in normal tissue, whereas in MCC tumors, the same genes were only in the top 5% to 10%. Of 32 patients with paired tumor and normal profiles, 9 (28.1%) had LOH at the HLA locus. Twenty-nine patients had copy number, LOH and expression data available for the HLA locus (online supplementary file 1). Six patients had LOH and less than half-maximal MHC class I gene expression, including four with less than median expression. Three patients with LOH also had a copy number gain. Trends for improved response and OS were observed in patients with higher vs lower MHC class I expression (figure 5). Trends were similar for HLA-A, HLA-B, HLA-C and overall MHC class I expression. MHC class I expression was higher in patients with median or greater CD8+ T cell density at the tumor invasive margin than in patients with less than median density (figure 5C). No trends were seen for CD8+ density at the tumor core.

Differential GSEA was performed in samples from 37 patients. As expected, pathways enriched in responders vs nonresponders included those associated with the inflammatory response (eg, interferon γ and interferon α/β), immune response (eg, Th1/Th2 pathway, natural killer (NK) T cells and Toll receptor pathways), and transforming growth factor-β signaling (online supplementary file 1). Gene sets associated with DNA replication and repair were enriched in nonresponders. Single-sample GSEA (ssGSEA) scores of gene sets for tumor necrosis factor α signaling via nuclear factor-κB, NK cell activation and the P53 pathway were highest in responders and patients with MCPyV− tumors (online supplementary file 1). For the interferon γ response gene set, scores were highest in responders and patients with MCPyV− or PD-L1+ tumors. In multifactorial ssGSEA analyses based on response and MCPyV or PD-L1 status, the greatest difference in score was for the P53 pathway between subgroups of nonresponders with MCPyV− or MCPyV+ tumors.

Biomarkers were also analyzed in the subgroup of patients with long-term OS (patients with >36-month OS after ≥44 months of follow-up; n=27). In 12 patients evaluable for TMB, the median TMB was 0.59 NSSV/Mb (range 0.18–31.62 NSSV/Mb), similar to that in the overall population. Of 22 patients evaluable for tumor PD-L1 expression, 18 (81.8%) had PD-L1+ tumors and 4 (18.2%) had PD-L1− tumors. Of 23 patients evaluable for MCPyV status, 13 (56.5%) had MCPyV+ tumors and 10 (43.5%) had MCPyV− tumors.

### Safety

After ≥36 months of follow-up, AEs of any grade had occurred in 86 of 88 patients (97.7%), of which 65 (73.9%) had a grade ≥3 AE. Treatment-related AEs (TRAEs) of any grade occurred in 68 patients (77.3%), which included six additional patients compared with a 10-month safety analysis.^10^ The most common TRAEs (>10%) were fatigue (22 (25.0%)), diarrhea (11 (12.5%)) and nausea (11 (12.5%); online supplementary file 1). Grade≥3 TRAEs occurred in 10 patients (11.4%; six additional patients since the 10-month analysis); those occurring in ≥1 patient were increased blood creatine phosphokinase (3 (3.4%)) and lymphopenia (2 (2.3%)). Nineteen patients (21.6%) had an irAE (online supplementary file 1), of which 4 (4.5%) had a grade ≥3 irAE (hypothyroidism, increased alanine aminotransferase, autoimmune disorder and increased transaminases). IRRs occurred in 19 patients (21.6%), none of which were grade ≥3. TRAEs led to discontinuation in eight patients (9.1%). No treatment-related deaths occurred. Of 13 patients who had received >52 doses and >2 years of avelumab treatment, 2 discontinued treatment because of a TRAE (suspected immune-related thrombocytopenia and immune-related colitis).

## Discussion

Chemotherapy offers limited benefits for patients with mMCC. In retrospective analyses of second-line or later chemotherapy, ORRs ranged from 10% to 23%, with no patient maintaining a response longer than 6 months, and 1-year PFS and OS rates were 0%.^4–6^ Avelumab was approved for the treatment of mMCC based on early results from the pivotal, phase 2 JAVELIN Merkel 200 trial, including durable responses in a subset of patients and a manageable safety profile, fulfilling an unmet medical need.^9^ With long-term follow-up, median DOR with avelumab monotherapy in previously treated patients with mMCC was 40.5 months, and a potential plateau in OS rates was observed (31% at 42 months). It should be noted that with ≥36 months of follow-up, the median duration of avelumab treatment remained relatively short (median 3.9 months; range 0.5–47.9 months), and only a small proportion of patients (6.8%) received >3 years of treatment. Although a proportion of patients had highly durable responses, disease progression occurred in approximately 40% of patients who had responded to avelumab. This rate of progression may be higher than that observed in studies of immune checkpoint inhibitors in metastatic melanoma,^19 20^ potentially reflecting the known highly aggressive nature of mMCC, although it should be noted that all patients in this study of avelumab had experienced disease progression with prior chemotherapy. Additionally, some patients remained on avelumab treatment after disease progression based on the investigator’s assessment of continued clinical benefit.

In biomarker analyses, clinical benefit was not consistently associated with any single biomarker. A trend for higher OS rates was seen in patients with PD-L1+ vs PD-L1− tumors, although 17% of patients were not evaluable for PD-L1 status. Most long-term survivors had PD-L1+ tumors, which suggests that although responses occurred irrespective of tumor PD-L1 status, patients with PD-L1+ tumors may have a higher probability of long-term OS. A potential trend for improved efficacy was also seen in patients with tumors with a high TMB, consistent with previous reports in MCC^21^ and other tumors,^22 23^ although fewer than half of patients (41%) were evaluable for TMB in this study. Response appeared to be more likely in tumors with high TMB that were also MCPyV−, or had a high CD8+ T cell density at the invasive margin or a high MHC class I expression level. Gene signature analyses suggested that pathways involved in NK cell activation were associated with response, consistent with a hypothesis that the antibody-dependent cell-mediated cytotoxicity activity of avelumab may contribute to clinical activity.^13 24^ Additional work is needed to further investigate these biomarker findings.

Limited data were available for outcomes with post-avelumab therapy. Although some patients had prolonged survival with subsequent therapy, most nonresponding patients died within months of discontinuing avelumab, consistent with the poor prognosis of mMCC. Further studies are needed to identify mechanisms of resistance to immune checkpoint inhibitors in mMCC and to evaluate novel treatment regimens to prolong OS in patients who have disease progression on immunotherapy or to increase the proportion of patients who respond initially.

The long-term safety profile of avelumab remained consistent with previous analyses^25^ and studies of other immune checkpoint inhibitors.^26–28^ No delayed safety signals or cumulative AEs were observed, and incidences of high-grade TRAEs and discontinuation due to TRAEs remained low.

The results reported here complement those from studies of anti–PD-1 agents in patients with MCC, including in earlier lines of therapy and disease stages. In the KEYNOTE-017 trial of pembrolizumab given as first-line therapy to 50 patients with stage IIIB or IV MCC, the ORR was 56% and median OS was not reached after a median follow-up of 14.9 months.^11^ In addition, in a cohort of 25 patients with stages II–IV MCC from the phase 1/2 CheckMate358 study of nivolumab, the ORR was 68% and median OS was not reached after 6.0 months of follow-up.^7^ Several ongoing trials are evaluating immune checkpoint inhibitors in combination with radiotherapy for patients with advanced MCC, or as monotherapy in earlier stages of disease. In particular, the randomized, placebo-controlled, phase 3 ADAM trial (NCT03271372) is investigating avelumab monotherapy as adjuvant treatment for patients with stage III MCC who have received definitive treatment (surgery and/or radiotherapy) for clinically detected metastases.

## Conclusions

With ≥44 months of follow-up for OS in this phase 2 trial, representing the longest prospective follow-up for a cohort of patients with mMCC reported to date, avelumab was well tolerated and showed continued durable responses and clinically meaningful survival outcomes in patients with mMCC, comparing favorably with historical studies of cytotoxic chemotherapy. These findings reinforce avelumab as a current standard-of-care treatment option for this patient population.
