# Supplementary material for: Avelumab in patients with previously treated metastatic Merkel cell carcinoma: long-term data and biomarker analyses from the single-arm phase 2 JAVELIN Merkel 200 trial
Source: J Immunother Cancer. 2020 May 15;8(1):e000674. doi: 10.1136/jitc-2020-000674 (PMC7239697; doi:10.1136/jitc-2020-000674)
Supplement: Supplementary data [file jitc-2020-000674supp001.pdf]

SUPPLEMENTARY MATERIAL

Table S1. Baseline characteristics

	**N=88**
Median age (range), years	72.5 (33-88)
Sex, n (%)	
Male	65 (73.9)
Female	23 (26.1)
ECOG PS, n (%)	
0	49 (55.7)
1	39 (44.3)
Site of primary tumor, n (%)	
Skin	67 (76.1)
Nonskin[a]	14 (15.9)
Missing	7 (8.0)
Visceral disease at study entry, n (%)	
Present	47 (53.4)
Absent	41 (46.6)
No. of prior systemic anticancer treatments, n (%)	
1	52 (59.1)
2	25 (28.4)
≥3	11 (12.5)
Tumor PD-L1 status, n (%)[b]	
Positive	57 (64.8)
Negative	16 (18.2)
Not evaluable	15 (17.0)
Tumor MCPyV status, n (%)	
Positive	46 (52.3)
Negative	31 (35.2)
Not evaluable	11 (12.5)

[a] Nonskin sites include lymph node (n=12 [13.6%]) and other sites (cheek mucosa and rectosigmoid junction; n=2 [2.3%]).

D'Angelo SP, *et al. J Immunother Cancer* 2020; 8:e000674. doi: 10.1136/jitc-2020-000674

[b] PD-L1+ status was defined as expression on ≥1% of tumor cells, assessed using a Dako PD-L1 73-10 IHC assay.

ECOG PS, Eastern Cooperative Oncology Group performance status; MCPyV, Merkel cell polyomavirus; PD-L1, programmed death-ligand 1.

Table S2. Summary of subsequent therapy received based on best overall response to avelumab

**Avelumab therapy (N=88)**		**Subsequent therapy[a]**					
**Best overall response[b]**	**Patients, n**	**Any reported therapy, n[a]**	**Radiotherapy or surgery**	**Chemotherapy agents**	**Immune checkpoint inhibitors[a]**	**Other systemic therapy**	**Investigator-reported patient status at data cutoff**
Complete response	10	3	Radiotherapy	–	–	–	Alive ≈12 months later
			Radiotherapy	Carboplatin plus etoposide	Nivolumab plus ipilimumab followed by nivolumab alone	–	Alive ≈3 years later
			Surgery	Carboplatin plus etoposide	–	–	Died within 2 months of starting chemotherapy
Partial response	19	3	Surgery	–	–	–	Alive >3 years later
			Radiotherapy	–	Pembrolizumab	Octreotide and pazopanib	Alive ≈2 year later
			–	Cyclophosphamide and capecitabine	–	–	Responded to chemotherapy but died ≈1 year later
No objective response	59 (SD=9; PD=32; NE=18)	21 (SD=2; PD=15; NE=4)	8 received radiotherapy	10 patients; agents included cisplatin, etoposide, topotecan, docetaxel, cyclophosphamide-doxorubicin, vincristine, capecitabine, and pegylated doxorubicin	5 patients; agents received were nivolumab and pembrolizumab	6 patients; agents included everolimus, somatostatin, sunitinib, and pazopanib	4 patients responded to chemotherapy; no other responses were recorded; 20 patients died; 1 patient was lost to follow-up

D'Angelo SP, *et al. J Immunother Cancer* 2020; 8:e000674. doi: 10.1136/jitc-2020-000674

[a] Excluding avelumab treatment.

[b] Tumor response according to RECIST 1.1 reported by independent review committee.

NE, not evaluable; PD, progressive disease; RECIST, Response Evaluation Criteria in Solid Tumors; SD, stable disease.

D'Angelo SP, *et al. J Immunother Cancer* 2020; 8:e000674. doi: 10.1136/jitc-2020-000674

Table S3. Treatment-related adverse events and infusion-related reactions (N=88)

	**Any grade, n (%)**	**Grade ≥3, n (%)**
Any treatment-related adverse event[a]	68 (77.3)	10 (11.4)
Fatigue	22 (25.0)	0
Diarrhea	11 (12.5)	0
Nausea	11 (12.5)	0
Rash	8 (9.1)	0
Asthenia	7 (8.0)	0
Decreased appetite	7 (8.0)	0
Hypothyroidism	6 (6.8)	1 (1.1)
Pruritus	6 (6.8)	0
Arthralgia	5 (5.7)	0
Aspartate aminotransferase increased	5 (5.7)	0
Blood creatine phosphokinase increased	5 (5.7)	3 (3.4)
Chills	5 (5.7)	0
Rash maculopapular	5 (5.7)	0
Alanine aminotransferase increased	4 (4.5)	1 (1.1)
Lymphopenia	3 (3.4)	2 (2.3)
Gamma glutamyltransferase increased	2 (2.3)	1 (1.1)
Autoimmune disorder	1 (1.1)	1 (1.1)
Blood cholesterol increased	1 (1.1)	1 (1.1)
Ileus	1 (1.1)	1 (1.1)
Neutrophil count decreased	1 (1.1)	1 (1.1)
Thrombocytopenia	1 (1.1)	1 (1.1)
Transaminases increased	1 (1.1)	1 (1.1)
Any infusion-related reaction[b]	19 (21.6)	0

Shown are individual treatment-related adverse events of any grade that occurred in >5% of patients and grade ≥3 treatment-related adverse events that occurred in any patient.

[a] The incidence of treatment-related infusion-related reaction based on the single Medical Dictionary for Regulatory Activities Preferred Term is not listed.

[b] Composite term, which includes adverse events categorized as infusion-related reaction,

anaphylactic reaction, drug hypersensitivity, type I hypersensitivity, or hypersensitivity reaction that occurred on the day of or day after infusion, in addition to signs/symptoms of infusion-related reaction that occurred on the day of infusion (during or after the infusion) that resolved on the day of onset or next day; includes adverse events classified by investigators as related or unrelated to treatment.

Table S4. Immune-related adverse events (N=88)

	**Any grade, n (%)**	**Grade ≥3, n (%)**
Any immune-related adverse event	19 (21.6)	4 (4.5)
Hypothyroidism	5 (5.7)	1 (1.1)
Rash	5 (5.7)	0
Diarrhea	2 (2.3)	0
Erythema	2 (2.3)	0
Abnormal thyroid function test	1 (1.1)	0
Alanine aminotransferase increased	1 (1.1)	1 (1.1)
Aspartate aminotransferase increased	1 (1.1)	0
Autoimmune disorder	1 (1.1)	1 (1.1)
Autoimmune colitis	1 (1.1)	0
Hemophagocytic lymphohistiocytosis	1 (1.1)	0
Hyperthyroidism	1 (1.1)	0
Pruritus	1 (1.1)	0
Rash maculopapular	1 (1.1)	0
Transaminases increased	1 (1.1)	1 (1.1)
Tubulointerstitial nephritis	1 (1.1)	0

D'Angelo SP, *et al. J Immunother Cancer* 2020; 8:e000674. doi: 10.1136/jitc-2020-000674

Table S5. Differentially expressed gene sets between responders and non-responders.

Rank	Gene pathway	P value	Adjusted P value	Enrichment score	Normalized enrichment score
**HALLMARK**					
1	HALLMARK_DNA_REPAIR	0.000197668	0.000460719	-0.415128008	-2.056632646
2	HALLMARK_COAGULATION	0.000198138	0.000460719	0.444763832	2.168012536
3	HALLMARK_OXIDATIVE_PHOSPHORYLATION	0.000199283	0.000460719	-0.447335279	-2.326239874
4	HALLMARK_TGF_BETA_SIGNALING	0.000199442	0.000460719	0.504282201	2.107973213
5	HALLMARK_EPITHELIAL_MESENCHYMAL_ TRANSITION	0.00019988	0.000460719	0.480098659	2.513865083
6	HALLMARK_IL2_STAT5_SIGNALING	0.00019988	0.000460719	0.383358989	2.007322367
7	HALLMARK_E2F_TARGETS	0.000200321	0.000460719	-0.424628781	-2.203893626
8	HALLMARK_MYC_TARGETS_V1	0.000200321	0.000460719	-0.603764626	-3.133638295
9	HALLMARK_INTERFERON_GAMMA_ RESPONSE	0.000200602	0.000460719	0.504527261	2.635977846
10	HALLMARK_INFLAMMATORY_RESPONSE	0.000201045	0.000460719	0.411378262	2.146191907
11	HALLMARK_ALLOGRAFT_REJECTION	0.000201045	0.000460719	0.427657346	2.231121136
12	HALLMARK_IL6_JAK_STAT3_SIGNALING	0.000201086	0.000460719	0.454104261	2.065831199
13	HALLMARK_MYC_TARGETS_V2	0.000201288	0.000460719	-0.69635539	-2.914081451
14	HALLMARK_UV_RESPONSE_DN	0.000201898	0.000460719	0.443026074	2.212918524
15	HALLMARK_COMPLEMENT	0.000201939	0.000460719	0.514428618	2.671397469
16	HALLMARK_INTERFERON_ALPHA_ RESPONSE	0.000202716	0.000460719	0.524541307	2.429599134
**Canonical pathways**					
1	REACTOME_METABOLISM_OF_PROTEINS	0.00019216	0.002428629	-0.478187531	-2.7025933
2	REACTOME_MRNA_PROCESSING	0.00019516	0.002428629	-0.424746945	-2.120428309
3	REACTOME_METABOLISM_OF_RNA	0.000195313	0.002428629	-0.559566834	-2.995237483
4	REACTOME_TRANSLATION	0.00019558	0.002428629	-0.740609769	-3.672384455
5	REACTOME_METABOLISM_OF_MRNA	0.000195656	0.002428629	-0.571411855	-2.979479908
6	REACTOME_PROCESSING_OF_CAPPED_ INTRON_CONTAINING_PRE_MRNA	0.000197006	0.002428629	-0.455673018	-2.231505359
7	REACTOME_INFLUENZA_LIFE_CYCLE	0.000197278	0.002428629	-0.68824442	-3.37141663
8	KEGG_SPLICEOSOME	0.000197394	0.002428629	-0.434990634	-2.101174493
9	REACTOME_NONSENSE_MEDIATED	0.000197394	0.002428629	-0.763746669	-3.600542435

	_DECAY_ENHANCED_BY_THE_EXON _JUNCTION_COMPLEX				
10	REACTOME_SRP_DEPENDENT_ COTRANSLATIONAL_PROTEIN_TARGETING_ TO_MEMBRANE	0.000197824	0.002428629	-0.759271896	-3.591056302
11	BIOCARTA_TH1TH2_PATHWAY	0.000197863	0.002428629	0.716116832	2.169325643
12	REACTOME_TCA_CYCLE_AND_ RESPIRATORY_ELECTRON_TRANSPORT	0.00019802	0.002428629	-0.502135659	-2.4010114
13	BIOCARTA_NKT_PATHWAY	0.000198177	0.002428629	0.690424832	2.37756372
14	PID_LYMPH_ANGIOGENESIS_PATHWAY	0.000198177	0.002428629	0.667236665	2.297712387
15	REACTOME_MRNA_SPLICING	0.000198216	0.002428629	-0.4786335	-2.251550708
16	KEGG_OXIDATIVE_PHOSPHORYLATION	0.000198491	0.002428629	-0.47162547	-2.243338859
17	PID_AVB3_INTEGRIN_PATHWAY	0.000198491	0.002428629	0.530411574	2.357838096
18	REACTOME_G_ALPHA1213_SIGNALLING_ EVENTS	0.000198491	0.002428629	0.50392102	2.240079659
19	KEGG_DNA_REPLICATION	0.00019857	0.002428629	-0.683602941	-2.555174011
20	REACTOME_FORMATION_OF_THE_ TERNARY_COMPLEX_AND_ SUBSEQUENTLY_THE_43S_COMPLEX	0.000198649	0.002428629	-0.795401936	-3.189087007
21	REACTOME_3_UTR_MEDIATED_ TRANSLATIONAL_REGULATION	0.000198728	0.002428629	-0.802572358	-3.764679226
22	KEGG_ALLOGRAFT_REJECTION	0.000198768	0.002428629	0.6943078	2.505486813
23	REACTOME_MITOCHONDRIAL_PROTEIN_ IMPORT	0.000198768	0.002428629	-0.639662913	-2.591950741
24	REACTOME_INFLUENZA_VIRAL_RNA_ TRANSCRIPTION_AND_REPLICATION	0.000199084	0.002428629	-0.781105699	-3.643800767
25	BIOCARTA_MCALPAIN_PATHWAY	0.000199164	0.002428629	0.623346924	2.095985925
26	REACTOME_RESPIRATORY_ELECTRON_ TRANSPORT	0.000199203	0.002428629	-0.533873359	-2.290658837
27	KEGG_RIBOSOME	0.000199243	0.002428629	-0.808036226	-3.661784274
28	KEGG_HEMATOPOIETIC_CELL_LINEAGE	0.000199283	0.002428629	0.495160832	2.209006628
29	PID_IL27_PATHWAY	0.000199442	0.002428629	0.609711084	2.07040902
30	REACTOME_INTERFERON_GAMMA _SIGNALING	0.000199442	0.002428629	0.580905213	2.43791162
31	KEGG_GRAFT_VERSUS_HOST_DISEASE	0.000199641	0.002428629	0.629004019	2.324186963
32	REACTOME_PEPTIDE_CHAIN_ELONGATION	0.000199641	0.002428629	-0.817409755	-3.694720109

D'Angelo SP, *et al. J Immunother Cancer* 2020; 8:e000674. doi: 10.1136/jitc-2020-000674

33	REACTOME_IL_3_5_AND_GM_CSF_SIGNALING	0.000199641	0.002428629	0.523697701	2.016587549
34	PID_INTEGRIN2_PATHWAY	0.000199681	0.002428629	0.604209218	2.139144877
35	REACTOME_INTEGRIN_CELL_SURFACE_INTERACTIONS	0.00019976	0.002428629	0.527056536	2.367069504
36	REACTOME_TRNA_AMINOACYLATION	0.00019984	0.002428629	-0.537688971	-2.098989198
37	PID_FAK_PATHWAY	0.00019988	0.002428629	0.53382316	2.248031234
38	REACTOME_ACTIVATION_OF_THE_MRNA_UPON_BINDING_OF_THE_CAP_BINDING_COMPLEX_AND_EIFS_AND_SUBSEQUENT_BINDING_TO_43S	0.00019988	0.002428629	-0.787961867	-3.256592689
39	REACTOME_RESPIRATORY_ELECTRON_TRANSPORT_ATP_SYNTHESIS_BY_CHEMIOSMOTIC_COUPLING_AND_HEAT_PRODUCTION_BY_UNCOUPLING_PROTEINS	0.00019988	0.002428629	-0.52070532	-2.335608839
40	NABA_BASEMENT_MEMBRANES	0.00019988	0.002428629	0.586705944	2.270338305
41	KEGG_TYPE_I_DIABETES_MELLITUS	0.00019992	0.002428629	0.561249149	2.128480389
42	PID_INTEGRIN1_PATHWAY	0.00020008	0.002428629	0.585528168	2.532211003
43	KEGG_BASE_EXCISION_REPAIR	0.00020028	0.002428629	-0.573882394	-2.110741572
44	KEGG_TGF_BETA_SIGNALING_PATHWAY	0.00020028	0.002428629	0.477643361	2.169167367
45	KEGG_AUTOIMMUNE_THYROID_DISEASE	0.00020028	0.002428629	0.67062933	2.43635355
46	KEGG_LEISHMANIA_INFECTION	0.000200361	0.002428629	0.544903459	2.365486029
47	REACTOME_CYTOSOLIC_TRNA_AMINOACYLATION	0.000200481	0.002428629	-0.636033718	-2.164682128
48	KEGG_ECM_RECEPTOR_INTERACTION	0.000200521	0.002428629	0.518252804	2.342513809
49	REACTOME_DNA_STRAND_ELONGATION	0.000200723	0.002428629	-0.701977021	-2.497948499
50	PID_CXCR4_PATHWAY	0.000200844	0.002428629	0.436336186	2.044197909
51	REACTOME_TOLL_RECEPTOR_CASCADES	0.000200965	0.002428629	0.427039261	2.031453624
52	REACTOME_EXTENSION_OF_TELOMERES	0.000200965	0.002428629	-0.673667957	-2.334311605
53	REACTOME_SIGNALING_BY_ILS	0.000201167	0.002428629	0.504513442	2.367854856
54	REACTOME_INTERFERON_ALPHA_BETA_SIGNALING	0.000201167	0.002428629	0.507011564	2.053460873
55	REACTOME_IMMUNOREGULATORY_INTERACTIONS_BETWEEN_A_LYMPHOID_AND_A_NON_LYMPHOID_CELL	0.000201288	0.002428629	0.511234307	2.156771341
56	KEGG_COMPLEMENT_AND_COAGULATION_	0.00020145	0.002428629	0.519220081	2.198203601

	CASCADES				
57	KEGG_NATURAL_KILLER_CELL_MEDIATED_CYTOTOXICITY	0.00020145	0.002428629	0.444060676	2.114916052
58	KEGG_TOLL_LIKE_RECEPTOR_SIGNALING_PATHWAY	0.000201491	0.002428629	0.471528624	2.152109725
59	PID_SYNDECAN_1_PATHWAY	0.000201572	0.002428629	0.591177589	2.360320923
60	KEGG_CELL_ADHESION_MOLECULES_CAMS	0.000202593	0.002428629	0.472488755	2.297982628
61	KEGG_CYTOKINE_CYTOKINE_RECEPTOR_INTERACTION	0.000202716	0.002428629	0.432329563	2.285437777
62	KEGG_JAK_STAT_SIGNALING_PATHWAY	0.000202799	0.002428629	0.469223201	2.262158489
63	REACTOME_INNATE_IMMUNE_SYSTEM	0.00020429	0.002428629	0.396035767	2.076510107
64	REACTOME_PLATELET_ACTIVATION_SIGNALING_AND_AGGREGATION	0.000205128	0.002428629	0.421544103	2.18321929
65	KEGG_FOCAL_ADHESION	0.000205339	0.002428629	0.410474678	2.132331484
66	REACTOME_HEMOSTASIS	0.00020829	0.002428629	0.364182633	2.076204388
67	REACTOME_SEMA4D_IN_SEMAPHORIN_SIGNALING	0.000397535	0.00382449	0.559296847	2.018284795
68	REACTOME_METABOLISM_OF_NON_CODING_RNA	0.000397772	0.00382449	-0.52005868	-2.095942393
69	PID_INTEGRIN_CS_PATHWAY	0.000397931	0.00382449	0.58139786	2.02307392
70	REACTOME_NOD1_2_SIGNALING_PATHWAY	0.000398406	0.00382449	0.572598612	2.04481269
71	PID_INTEGRIN3_PATHWAY	0.000399122	0.00382449	0.52660652	2.075985385
72	PID_TCPTP_PATHWAY	0.00040016	0.00382449	0.531531303	2.082235242
73	KEGG_RNA_POLYMERASE	0.000401445	0.00382449	-0.576137245	-2.050154241
74	REACTOME_BASE_EXCISION_REPAIR	0.000601805	0.005213431	-0.628341356	-2.003157361
75	NABA_COLLAGENS	0.00060241	0.005213431	0.511876411	2.023622852
76	REACTOME_LAGGING_STRAND_SYNTHESIS	0.0006035	0.005213431	-0.643059034	-2.022357217
77	BIOCARTA_UCALPAIN_PATHWAY	0.001192606	0.008723965	0.646166142	2.024635975

A positive enrichment score identifies gene sets enriched in responders (PR/CR), a negative enrichment score identifies gene sets enriched in nonresponders (SD/PD).

Figure S1. Study profile after ≥44-months of follow-up.

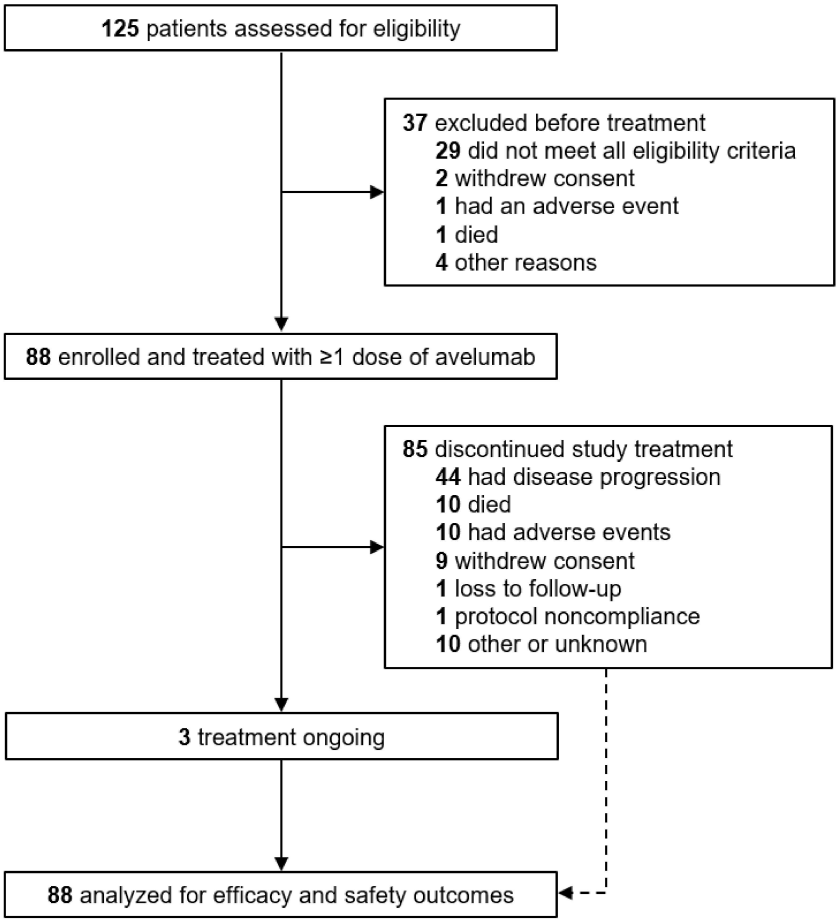

D'Angelo SP, *et al. J Immunother Cancer* 2020; 8:e000674. doi: 10.1136/jitc-2020-000674

Figure S2. Best change from baseline in target lesions in evaluable patients (patients with a baseline tumor assessment and ≥1 on-study tumor assessment; n=65).

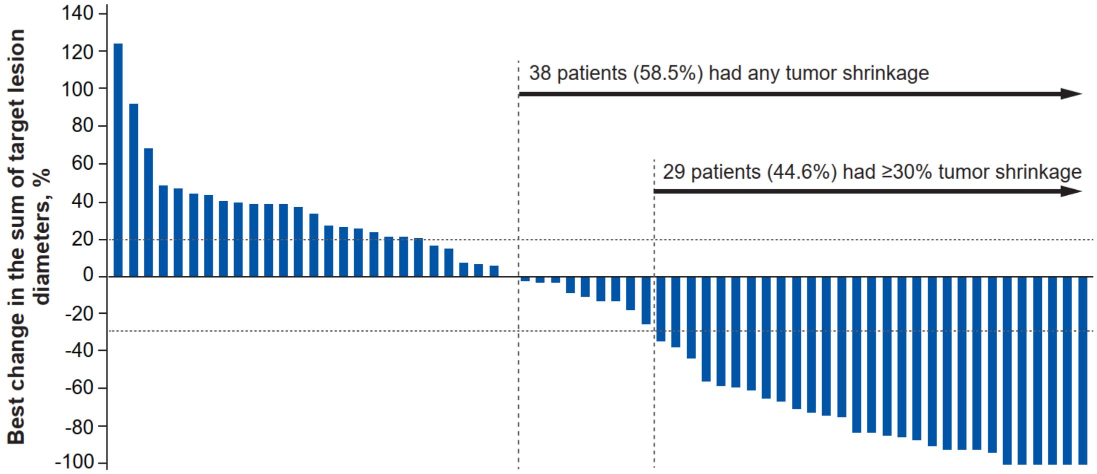

D'Angelo SP, *et al. J Immunother Cancer* 2020; 8:e000674. doi: 10.1136/jitc-2020-000674

Figure S3. Change from baseline in target lesions over time by tumor PD-L1 expression in evaluable patients who had a confirmed response to avelumab (n=26). PD-L1, programmed death-ligand 1.

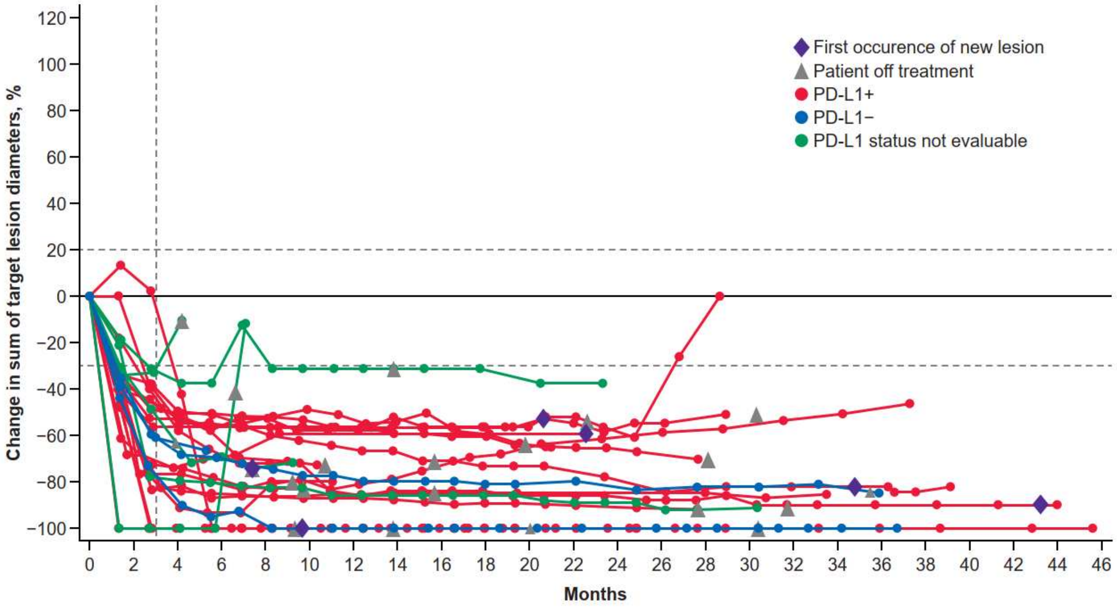

D'Angelo SP, *et al. J Immunother Cancer* 2020; 8:e000674. doi: 10.1136/jitc-2020-000674

Figure S4. HLA gene expression in (A) MCC tumor samples and normal tissues; (B) genomic changes in MHC class I HLA genes from evaluable patients (n=29). The boxes represent interquartile ranges, and the horizontal lines are medians. The whiskers denote the lower and upper quartiles, and the circles represent data points. CN, copy number; CPM, counts per million; CR, complete response; GTEx, Genotype-Tissue Expression; LOH, loss of heterozygosity; MCC, Merkel cell carcinoma; MHC, major histocompatibility complex; NE, not evaluable; PD, progressive disease; PR, partial response; SD, stable disease; TPM, transcripts per kilobase per million.

A

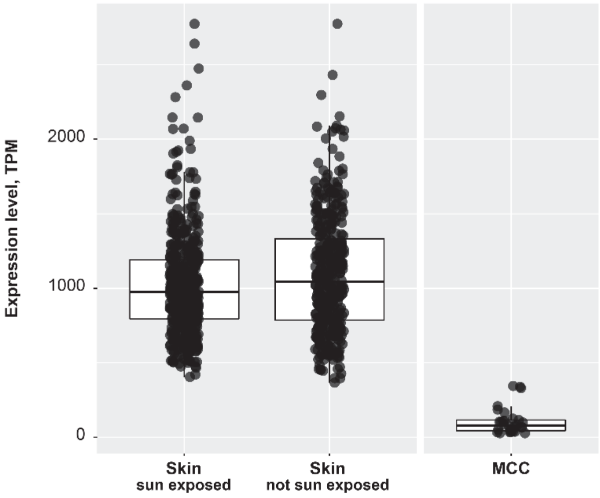

Expression rank, n (%)	MCC	GTEx.Skin	GTEx.Overall
**HLA-A**	1927 (9.66)	86 (0.2)	92 (0.17)
**HLA-B**	1121 (5.62)	44 (0.1)	36 (0.07)
**HLA-C**	1710 (8.57)	102 (0.24)	86 (0.16)

D'Angelo SP, *et al. J Immunother Cancer* 2020; 8:e000674. doi: 10.1136/jitc-2020-000674

B

Patient index	CN	LOH	CPM	Response
1	3		15.16	PR
2	3	Yes	32.77	NE
3	2		34.96	SD
4	2	Yes	46.01	NE
5	4		47.86	SD
6	2		48.96	SD
7	2	Yes	69.2	PD
8	2		71.05	NE
9	2		82.76	PD
10	3		121	PR
11	2		140.22	PD
12	2		156.88	PD
13	2		157.7	PD
14	2	Yes	160.83	PD
15	2		170.15	PD
16	2		182.03	PD
17	2		229.94	CR
18	2		233.74	PD
19	2		236.49	NE
20	3	Yes	267.41	PD
21	2		282	PD
22	2		308.44	PR
23	2		311.39	SD
24	4		347.03	PR
25	0		379.35	PD
26	3	Yes	545.24	CR
27	2		765.3	PR
28	2		1216.92	PR
29	2		1225.24	SD

D'Angelo SP, *et al. J Immunother Cancer* 2020; 8:e000674. doi: 10.1136/jitc-2020-000674

Figure S5. Differential gene set enrichment and association with biomarker status and clinical response for (A) tumor necrosis factor α signaling via NF-κB, (B) NK cell activation, (C) interferon γ response, and (D) P53 pathway. The boxes represent interquartile ranges, and the horizontal lines are medians. The whiskers denote the lower and upper quartiles, and the circles represent data points. MCPyV, Merkel cell polyomavirus; NF-κB, nuclear factor–κB; NK, natural killer; NR, nonresponders; PD-L1, programmed death-ligand 1; R, responders.

A

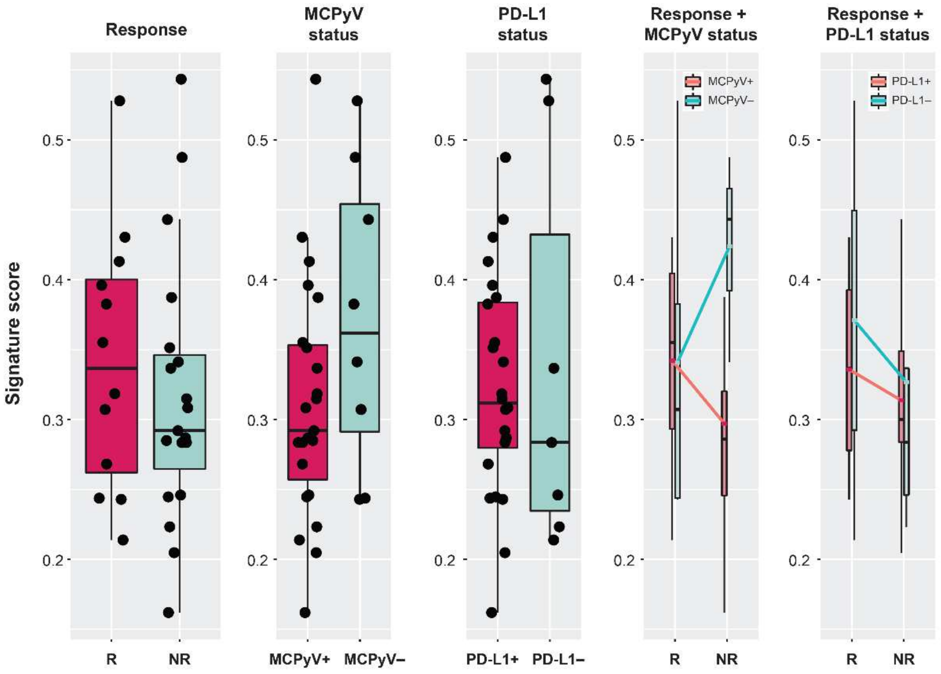

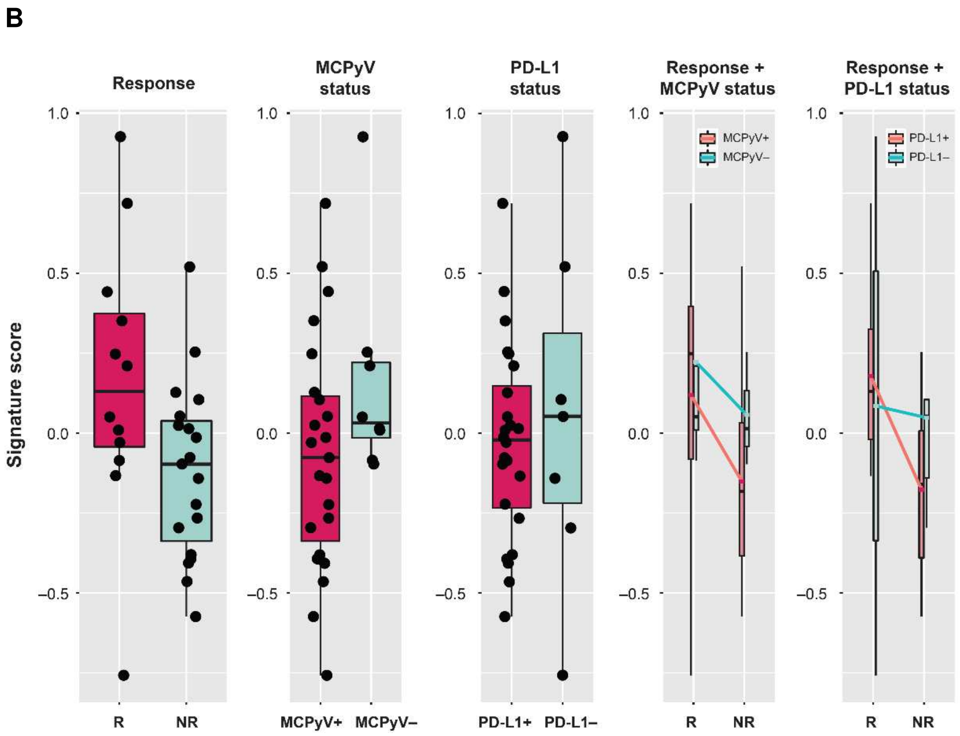
B
Response
MCPyV status
PD-L1 status
Response + MCPyV status
Response + PD-L1 status
Signature score
1.0
0.5
0.0
−0.5
R
NR
MCPyV+
MCPyV−
PD-L1+
PD-L1−

C

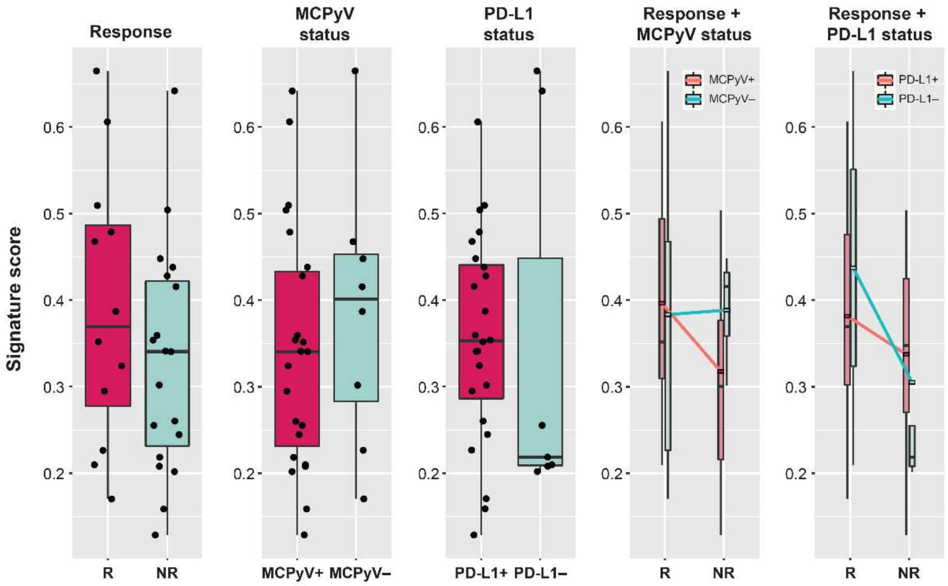
Response
MCPyV status
PD-L1 status
Response + MCPyV status
Response + PD-L1 status
Signature score
0.6
0.5
0.4
0.3
0.2
R
NR
MCPyV+
MCPyV–
PD-L1+
PD-L1–

D

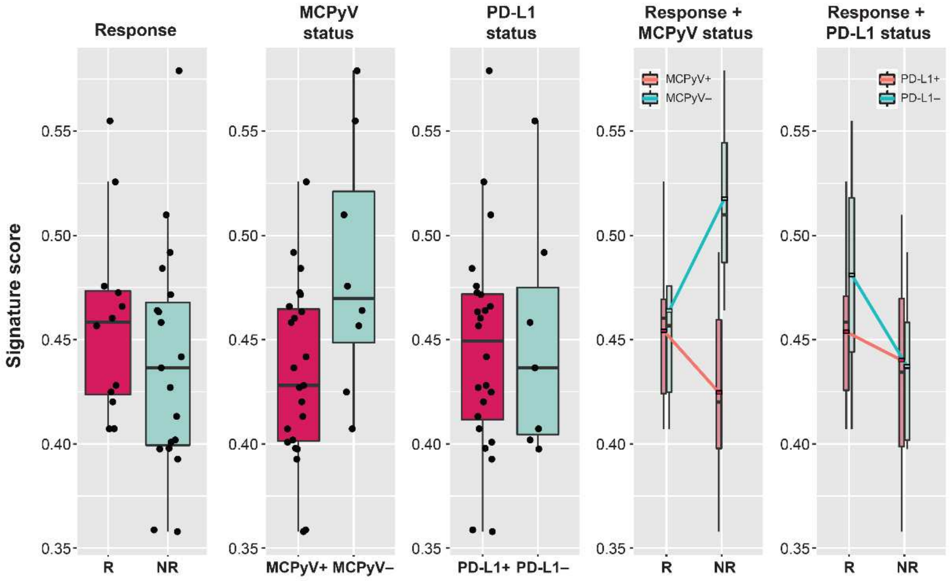
Response
MCPyV status
PD-L1 status
Response + MCPyV status
Response + PD-L1 status
Signature score
0.55
0.50
0.45
0.40
0.35
R
NR
MCPyV+
MCPyV–
PD-L1+
PD-L1–
